# Screening of Deoxynivalenol Producing Strains and Elucidation of Possible Toxigenic Molecular Mechanism

**DOI:** 10.3390/toxins9060184

**Published:** 2017-06-01

**Authors:** Xiangfeng Zheng, Xiaoli Zhang, Lina Zhao, Maurice T. Apaliya, Qiya Yang, Wei Sun, Xiaoyun Zhang, Hongyin Zhang

**Affiliations:** 1 School of Food and Biological Engineering, Jiangsu University, Zhenjiang 212013, Jiangsu, China; xiangfengzheng@126.com (X.Z.); zhangxiaoli0301@126.com (X.Z.); linabobo0706@163.com (L.Z.); mtapaliya@yahoo.com (M.T.A.); yangqiya1118@163.com (Q.Y.); zhangxiaoyungu@126.com (X.Z.); 2 Zhen Jiang Grain and Oil Quality Testing Center, Zhenjiang 212013, Jiangsu, China; lsj315@126.com

**Keywords:** deoxynivalenol, *Fusarium graminearum*, proteomics, transcriptomics

## Abstract

In this study, seven strains of *Fusarium graminearum* were isolated from wheat, of which six were identified to produce deoxynivalenol and the production of deoxynivalenol was assessed. *F. graminearum* strain Fg1 was noted to produce 1.0 μg/g deoxynivalenol during the incubation period in the Czapek yeast broth, while none was detected in *F. graminearum* strain Fg2. Hence, the differences in proteomes and transcriptomes of Fg1 and Fg2 were compared to analyze the mechanism underlying deoxynivalenol production. Among the 66 significantly differentially expressed proteins in Fg1, 39 and 27 were more or less abundant expressed. Functional analysis suggested that the enzymes involved in the methylerythritol 4-phosphate and mevalonate pathways, which provide a substrate for biosynthesis of farnesyl pyrophosphate, a precursor of DON, were activated in Fg1. The transcriptomics data demonstrated that the expression level of a majority of genes, including trichothecene biosynthetic genes, protein kinases, and transcription factors, involved in trichothecene biosynthesis was higher in Fg1 than in Fg2. The results also revealed differential expression profiles of deoxynivalenol biosynthesis genes in strains Fg1 and Fg2, which emphasized their deoxynivalenol producing ability and the underlying mechanism.

## 1. Introduction

Deoxynivalenol (DON) is a secondary metabolite mainly produced by *Fusarium* species, such as *F. Graminearum* and *F. culmorum*. DON is commonly found in food and feed. A three-year survey conducted by Rodrigues and Naehrer (2012) indicated that 79% of corn and 76% of wheat samples were contaminated with DON in North America [1]. In the southern region of Brazil, 243–2281 μg of DON/kg whole wheat samples was detected in 2012 [2]. A recent study revealed that DON was the major toxin found in wheat bran, corn, and soybean meal, in Chinese markets [3]. Gratz and Garcia reported that the level of DON contamination in food is greatly affected by weather and farming conditions [4,5].

DON is also known as vomitoxin because of its effects on livestock; it exerts toxic effects in humans and domestic animals. It can induce apoptosis of various cells, and is well-known for its neurotoxicity, genotoxicity, immunotoxicity, mutagenesis and misshapen effects [6]. Toxicity studies revealed that DON inhibits eukaryotic protein synthesis, which disrupts cytokine regulation and alters cell proliferation, leading to cell death [7]. DON was also shown to combine with protein sulfhydryl groups and inhibits mitochondrial protein synthesis, leading to the induction of oxidative stress [8].

In recent years, several trichothecene biosynthetic genes (*TRI*) revealed in DON biosynthesis were revealed [9]. Until now, 15 highly conserved genes were found to be closely associated with biosynthesis of *Fusarium* trichothecene toxins [10]. These genes are scattered over different chromosomes, namely *TRI5* cluster, *TRI1* and *TRI16* double cluster and *TRI101* [10]. The *TRI5* core cluster consists of 12 genes (*TRI3*-*TRI14*), which are closely related to *TRI5* and are arranged in a DNA fragment of about 30 kb. The *TRI5* core cluster encodes several proteins, such as trichodiene synthase (*TRI5*) [11], P450 monooxygenase (*TRI4*, *TRI11*, *TRI1*3) [12], trichothecenes C-3 esterase (*TRI8*) [13], 15-O-acetyltransferase (*TRI3*) [14], transcription factors (*TRI6*, *TRI10*) [15], toxin delivery pump (*TRI12*) [16], 4-O-acetyltransferase (*TRI7*) and two unknown functional proteins (*TRI9* and *TRI14*). Terpene cyclase gene *TRI5* isolated from *F. sporotrichioides* has been reported to be involved in DON synthesis [17,18]. *TRI1* encodes cytochrome P450 monooxygenase in *F. sporotrichioides* and *F. graminearum* [19,20]. *TRI16* encodes acyltransferase and catalyses the formation of ester side groups at C-8 during trichothecene biosynthesis in *F. sporotrichioides* [21]. The *TRI101* encodes 3-O-acetyltransferase, which is able to convert the toxin into a less toxic product [22]. 

The production of DON is regulated not only by *TRI*, but also by protein kinases [23]. There are about 120 predicted protein kinase genes in *F. graminearum*. Ochiai reported that the expression level of *TRI4* and *TRI6* in the *F. graminearum* mutants-*FgOs4* (encoding mitogen-activated protein kinase kinase kinase; MAPKKK), *FgOs5* (encoding mitogen-activated protein kinase kinase; MAPKK), and *FgOs2* (encoding mitogen-activated protein kinase; MAPK) were markedly reduced in rice medium [24]. In addition, the toxigenic capability of these mutants was blocked [24]. Target of rapamycin (TOR) is also a serine/threonine kinase, which plays an important role in cell growth, proliferation, metabolism, protein translation and signaling pathways [25,26,27]. Yu et al. found that *FgPPG1* (FGSG_05281) mutants showed a decrease of sporulation and failure to produce DON; *FgPPG1* is one of the key TOR genes in *F. graminearum* [26]. In addition, FgPPG1 interacts with FgTip41 (FGSG_06963) to regulate mycelia growth and virulence. It also regulates DON synthesis and *F. graminearum* pathogenicity by regulating downstream transcription factor FgAreA (FGSG_08634) [26]. *FgAtf1* is a transcription factor that plays a negative regulatory effect on DON synthesis [28].

The molecular mechanism underlying DON biosynthesis in *F. graminearum* has almost been revealed. However, in the current study, DON-producing capacities of Fg1 and Fg2 strains were stuided. Thus, (i) Expression level of the genes involved in DON synthesis was analyzed by proteomics and transcriptomics analyses of a DON-producing strain, Fg1, and DON non-producing strain, Fg2 and (ii) the possible molecular mechanism underlying DON production by Fg1 was investigated.

## 2. Results

### 2.1. Screening and Identification of F. graminearum

**Figure 1:**
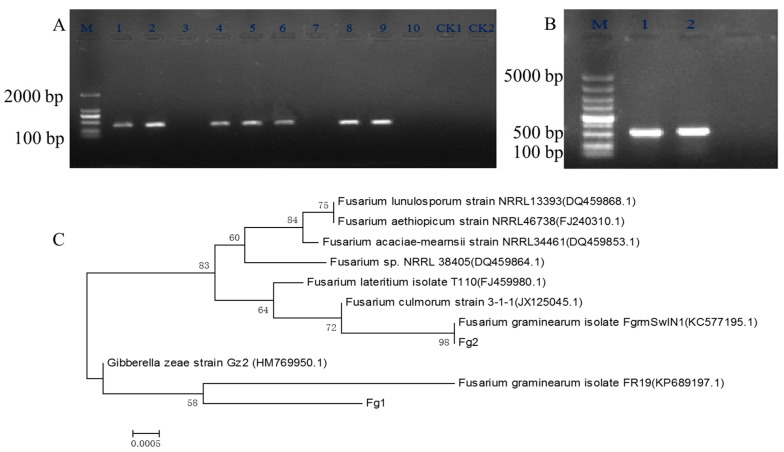
The electrophoresis of *Fusarium* spp. using primer Fg16F/Fg16R, ITS1/ITS4 and the phylogenetic tree of the ITS domain sequences of *F. graminearum* Fg1 and Fg2. (**A**) The PCR result of *Fusarium* spp. using Fg16F/Fg16R as the specific primer. M: DNA Maker, 1–10 lanes are for the 10 strains of *Fusarium* spp., Lane 11 and 12 are as the negative control strains; (**B**) The PCR result of *F. graminearum* using ITS1 and ITS4 as the primers; (**C**) The phylogenetic tree of Fg1 and Fg2 strain.

Ten strains of *Fusarium*, spp. were isolated from wheat grain which was infected by Fusarium head blight, and were screened by specific PCR using genomic DNA as a template and Fg16F/Fg16R as primers. From the PCR analysis, among the 10 strains, 7 were identified to be *F. graminearum*, Figure 1A. The capacity of DON production of these 7 *F. graminearum* strains was studied under similar growth conditions. The results showed that Fg1 produced 1.02 μg/g of DON, Table 1, while Fg2 did not produce DON. Hence, Fg1 and Fg2 strains were identified by further studying the internal transcribed spacer (ITS) region. The DNA product was purified from the gel and sequenced, Figure 1B. The phylogenetic tree with the homologous species which was acquired by BLAST analysis of the ITS region using NCBI nr database, was built. As shown in Figure 1C, both Fg1 and Fg2 were identified to be different strains of *F. graminearum*. 

### 2.2. Identification of Differentially Expressed Proteins in Fg1 and Fg2 Strains

**Figure 2:**
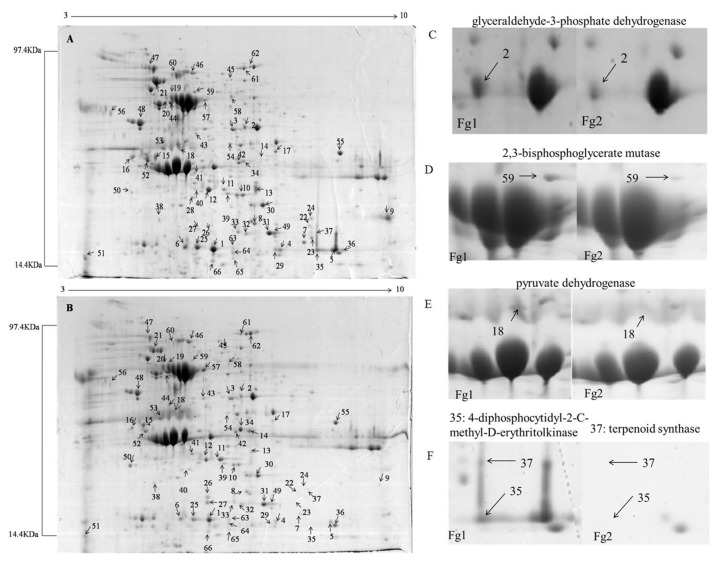
Proteome analysis of the strain Fg1 and Fg2. (**A**) Proteome analysis of Fg1; (**B**) Proteome analysis of Fg2; (**C**–**F**) represent the protein spot of 2, 59, 18 and 35, 37, respectively.

Figure 2A,B show two dimensional gels of total protein extracted from Fg1 and Fg2. A total of 152 differentially expressed (average fold change ≥ 1.5) proteins were detected in each gel. Among them, 66 proteins were significantly differentially expressed (Average fold change ≥ 2, *p* < 0.05), out of which, 39 were significantly up-regulated (Fg1/Fg2 ≥ 2, *p* < 0.05), while 27 were significantly down-regulated (Fg1/Fg2 ≤ 0.5, *p* < 0.05). Additionally, 48 of the best-resolved protein spots which were significantly differentially expressed, were identified by mass spectrometry (MS). Detailed information about lowercase letters in peptide names is given in Table 2. It was noticed that expression of the protein at spot 2 (glyceraldehyde-3-phosphate dehydrogenase), Figure 2C, spot 59 (2,3-bisphosphoglycerate mutase, Figure 2D), spot 18 (pyruvate dehydrogenase, Figure 2E), and spot 37 (terpenoid synthase, Figure 2F), was significantly up-regulated in Fg1, which were 6.21, 3.53, 7.02 and 10.18 times higher than that in Fg2, respectively.

### 2.3. Gene Ontology (GO) Analysis and Function Classification of Differentially Expressed Proteins in Fg1 and Fg2 Strains

**Figure 3:**
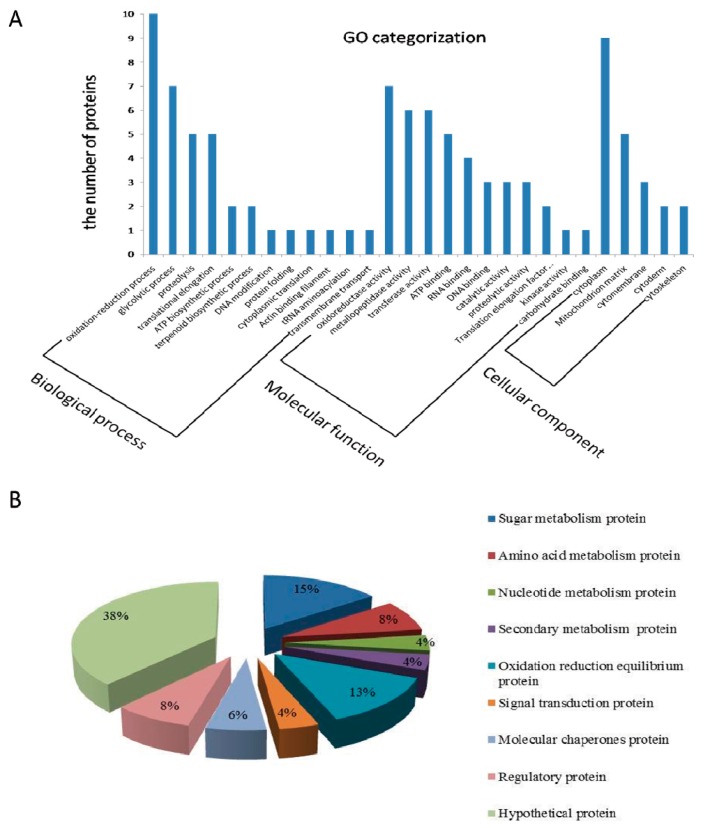
(**A**) GO categorization, and (**B**) function classification of differentially expressed proteins in Fg1 and Fg2 strains.

Forty-eight differentially expressed proteins were classified to the category of cell component, biological process and molecular function, and among them, 12 were observed to be classified under biological process, Figure 3A. Most of them were involved in oxidation-reduction, glycolytic process, proteolysis and translational elongation. Under the molecular function, the results revealed that seven proteins were involved in oxidoreductase activity, and six proteins each were involved in metalloproteinase and transferase activities, respectively. In case of the cellular component, most of the differentially expressed proteins were classified in the cytoplasm. Five proteins were located in the mitochondria, and a few proteins were located in the cell membrane and cytoskeleton.

The proteins were categorized according to their functions, Figure 3B. Most of them were hypothetical proteins. Figure 3B shows that 15% of the proteins were involved in glucose metabolism, and 13% in oxidation reduction equilibrium. The proportion of amino acid metabolic and regulatory proteins was 8% each.

### 2.4. mRNA-Seq and GO Analysis of the Different Expressed Genes of Fg1 and Fg2

**Figure 4:**
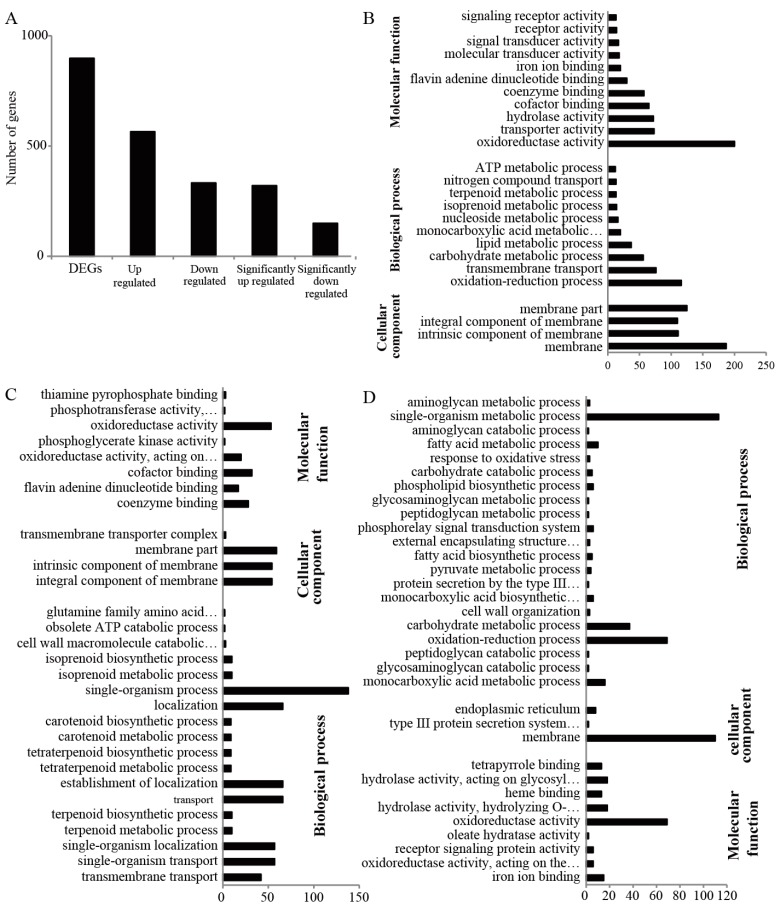
Bioinformatics analysis of the RNA-seq result. (**A**) Toatl number of DEGs between Fg1 and Fg2; (**B**) GO analysis of the DEGs; (**C**) GO analysis of the down-regulated genes in Fg1; (**D**) GO analysis of the up-regulated genes in Fg1.

The transcriptomes of Fg1 and Fg2 were analyzed using mRNA-Seq, and RNA-seq libraries were sequenced on an Illumina HiSeq 2000 system. Totally, 26363446 and 31301746 clean reads were acquired in Fg1 and Fg2, respectively. Table S1. The reads were mapped to the reference genome of *F. graminearum* PH-1(taxid: 229533). Totally, 81.36% and 78.06% of the reads of Fg1 and Fg2, respectively, were mapped to the reference genome, Table S2. The reads were assembled and the expression level was evaluated, and 898 differentially (|log2 Fg1/Fg2| > 1) expressed genes were detected. In Fg1, expression levels of 333 genes were down-regulated (log2 Fg1/Fg2 < −1), while those of 565 genes were up-regulated as compared to those in Fg2 (log2 Fg1/Fg2 > 1), Figure 4A. Among the differentially expressed genes (DEGs), 321 were significantly up-regulated (log2 Fg1/Fg2 > 2), and 150 were significantly down-regulated (log2 Fg1/Fg2 < −2) in Fg1 as compared to those in Fg2, Figure 4A. 

The GO enrichment classification of DEGs is shown in Figure 4B. From the results, the 898 DEGs were classified into 3 main groups, which were subdivided into 25 subgroups. Under the molecular function, DEGs involved in oxidoreductase activity were the highest followed by those involved in transport activities, hydrolase activities, cofactor and coenzyme binding activities. Consistent with the molecular function, most of the DEGs were involved in oxidation-reduction process. It was also observed that transmembrane transport, carbohydrate metabolism and lipid metabolism processes accounted for a large proportion of the biological process. Although all the DEGs were located in the cell membrane, they were classified as membrane, intrinsic and integral component of the membrane.

GO analysis was also performed on both down-regulated and up-regulated proteins. As shown in the Figure 4C,D, the down-regulated proteins in Fg1 were mainly involved in oxidoredutase activity. Most of the down-regulated genes, which were involved in single-organism process, localization, establishment of localization transport, single-organism localization and single-organism transport under the biological process classification, were located at the membrane Figure 4C. Most of the up-regulated genes were involved in oxidoreductase activity and located at the membrane. Under the biological process, most of the genes were classified under single-organism metabolic process followed by oxidation-reduction and carbohydrate metabolic processes, Figure 4D.

### 2.5. Kyoto Encyclopedia of Genes and Genomes Pathway Analysis of DEGs of Fg1 and Fg2 Strains

**Figure 5:**
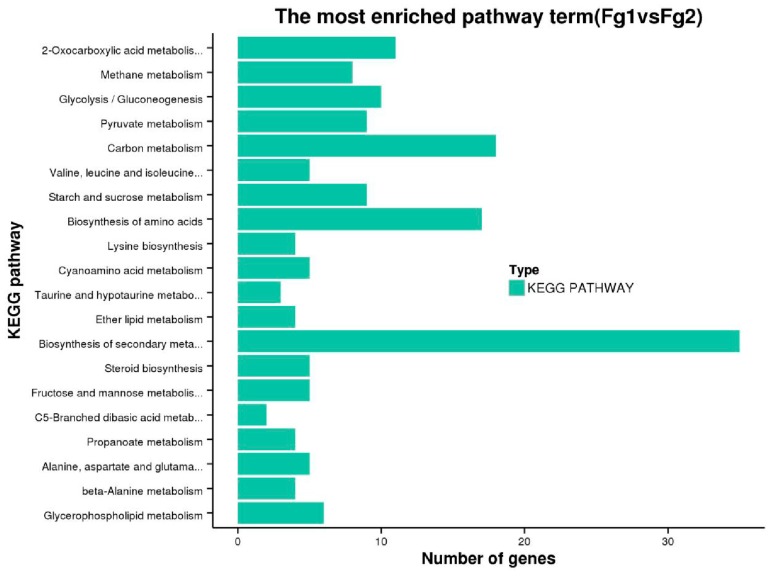
The most enriched KEGG pathway of the DEGs of Fg1 and Fg2 strain.

The DEGs were grouped into 68 Kyoto Encyclopedia of Genes and Genomes (KEGG) pathways through the KEGG database. To analyze the functions of DEGs of the two strains involved in the metabolic pathway, we selected the 20 most significant pathways, Figure 5. The highest metabolic pathway was the biosynthesis of secondary metabolites, containing 35 DEGs. The result also revealed that 18 DEGs were involved in carbon metabolism pathway, 17 were involved in amino acid biosynthesis, and 3 were involved in pantothenic acid and coenzyme A biosynthesis.

### 2.6. The Expression Level of Genes Involved in DON Biosynthesis in Fg1 and Fg2

**Figure 6:**
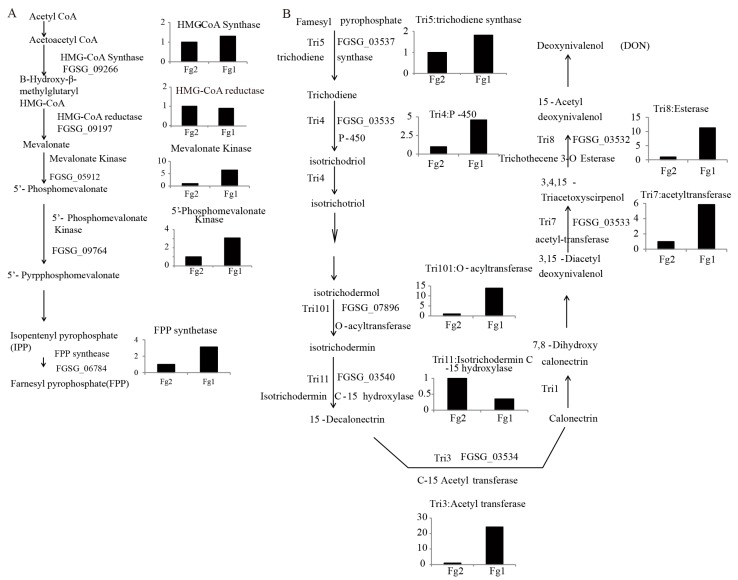
The biosynthesis pathway of DON in *F. graminearum*. (**A**) The synthesis pathway of FPP; (**B**) The synthesis pathway of DON. Gene symbol was marked as FG_*****, gene annotation was shown with the name. Gene expression level represents the data from the RNA-seq.

Farnesyl pyrophosphate (FPP) is the main substrate of DON biosynthesis. As shown in Figure 6A, the expression level of FGSG_09266 (HMG-CoA Synthase) and FGSG_09197 (HMG-CoA reductase) was not significantly different between Fg1 and Fg2. However, the genes located downstream of FPP biosynthesis genes were all up-regulated in Fg1. The expression levels of FGSG_05912 (mevalonate [MVA] kinase), FGSG_09764 (5′-phosphoMVA) and FGSG_06784 (FPP synthase) in Fg1 were 5, 3 and 3 times higher than those in the Fg2, respectively. The expression of these genes involved in DON synthesis was also analyzed, Figure 6B. Seven genes involved in DON synthesis were compared, out of which 6 were significantly up-regulated in Fg1, Figure 6B. The expression level of FGSG_03537 and FGSG_03535 in Fg1 was 1.5 and 4 times higher than that of these genes in Fg2, respectively. The expression level of FGSG_7896, FGSG_03532 and FGSG_03533 in Fg1 was 5 times higher than that of these genes in Fg2.

### 2.7. Verification of the Gene Expression Level by Real Time PCR

**Figure 7:**
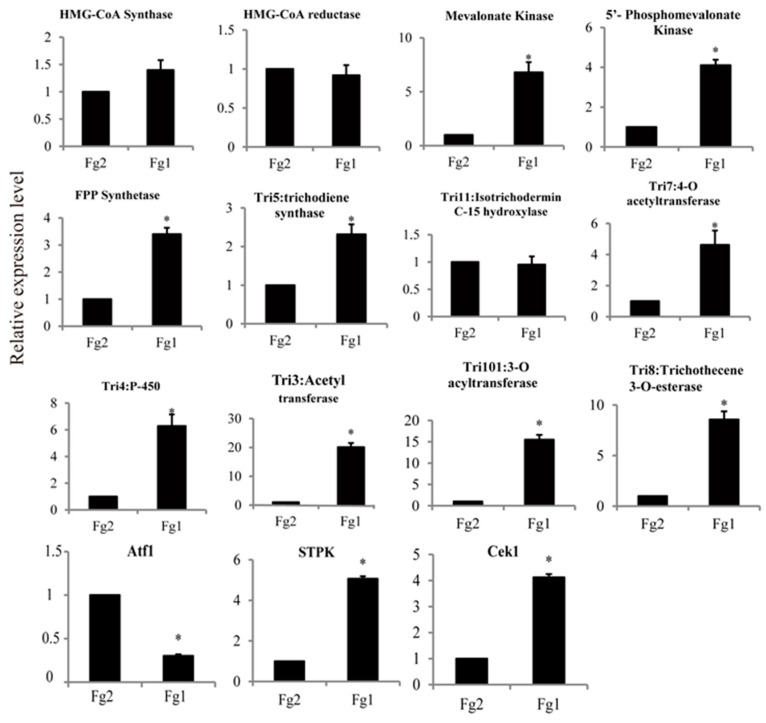
Verification of the different expressed genes which involved in DON synthesis. * Represent significantly difference in their expression level (*p* < 0.05).

The expression level of 12 genes involved in DON biosynthesis was verified by real time PCR (qPCR). From the result of RNA-seq, the expression level of FGSG_09266 (HMG-CoA Synthase), FGSG_09197 (HMG-CoA reductase) and FGSG_03540 (Tri11: isotrichodermin C-15 hydroxylase) was not significantly different. However, all the other genes were significantly up-regulated in Fg1, Figure 7. Expression level of these three genes, which encode MVA kinase, 5′-phosphoMVA kinase and FPP synthase, respectively, was significantly increased in Fg1, and was 6.88, 4.03 and 3.37 times higher than that of these genes in Fg2, respectively. In addition, expression level of *TRI5*, *TRI7*, *TRI4*, *TRI3* and *TRI8* was also significantly up regulated in Fg1, and was 2.38, 4.79, 6.42, 20.92 and 8.78 times hegher than that of these genes in Fg2, respectively. These genes were located at the *TRI5* core cluster. The expression of another gene, *TRI101*, which encodes 3-O-acyltransferase, was 10 times in Fg1 than in Fg2. However, the expression level of the negative regulation transcription factor, *Atf1*, was much lower in Fg1 than in Fg2. In addition, the expression levels of serine-threonine protein kinase (STPK) and STPK-Cek1 were 5.06 and 4.12 times higher in Fg1 than that in Fg2, respectively. 

## 3. Discussion

In this study, we used two strains—Fg1, DON-producing strain and Fg2, DON non-producing—to analyze the molecular mechanism of DON biosynthesis. The results on the proteomics analysis of the strains revealed 152 differentially expressed proteins, among which, 48 were identified. The GO and functional classification analyses showed that most of the proteins were involved in oxidation-reduction and cell-based metabolism processes. Similarly, the GO analysis performed using the transcriptome data demonstrated that most of the DEGs were classified under oxidation-reduction process, and a positive correlation was observed between the findings of the proteomics and transcriptomics analyses. The results of the transcriptomics analysis revealed that the gene expression profile was different in Fg1 and Fg2. These results demonstrated the difference in phenotypes considering DON production. Hestbjerg et al., (2002) showed a significant correlation between DON concentration and disease index [29]. However, a majority of the down-regulated genes were involved in localization, establishment of localization and single-organism localization in Fg1. Maier et al. (2006) also reported that DON is pathogenicity factor, which may influence the virulence of *F. graminearum* in a complex manner [30].The results indicated that the pathogenicity of Fg1 may be weaker than that of Fg2, leading to the production of higher amount of toxin influencing the virulence of Fg1. 

DON is a derivative of sesquiterpene compounds, which are synthesized by FPP, which is synthesized through MVA and methylerythritol 4-phosphate (MEP)-independent pathways. Pyruvate and glyceraldehyde-3-phosphate are the main substrates of the MEP pathway. Glyceraldehyde-3-phosphate dehydrogenase (protein spots 2, 4 and 8) and 2,3-bisphosphoglycerate mutase (protein spot 59) are the key enzymes required for the production of pyruvate and glyceraldehyde-3-phosphate [31]. The expression level of these enzymes was significantly higher in Fg1 than in Fg2. The expression level of these two genes followed the same trend. The biosynthesis of FPP can also be achieved through the MVA pathway, which uses acetyl CoA as the only substrate [32]. Pyruvate dehydrogenase (PDH, spot 18) is the key enzyme responsible for transforming pyruvate to acetyl CoA, and providing important raw material for FPP biosynthesis [33]. The level of PDH expression in Fg1 was significantly higher in Fg1 than in Fg2. In addition, the gene annotated to MVA kinase, 5′-PhosphoMVA and MVA 5-diphosphate were all significantly up-regulated in Fg1. These findings indicated that both MEP and MVA pathways were activated in Fg1, which provided more substrate for biosynthesis of FPP, which is a precursor of DON.

KEGG enrichment analysis revealed that the most enriched pathway was biosynthesis of secondary metabolites. The expression level of secondary metabolism-related proteins was significantly different in the two strains as demonstrated by the levels of 4-diphosphocytidyl-2-C-methyl-D-erythritolkinase (CMK, spot 35) and terpenoid synthase (TPS, spot 37), Figure 2F. CMK, one of the key enzymes involved in natural terpenoid biosynthetic pathway, can trigger 4-(5-pyrophosphate cytidine)-2-C-methyl-D-erythritol derivative phosphate to generate 4-(5-pyrophosphate cytidine)-2-C-methyl-D-erythritol 2-phosphate [34]. Therefore, it regulates the synthesis of terpene compounds.

Terpene synthase is a key enzyme associated with “carbon flux”, which controls terpene biosynthetic pathway, and it can catalyze the conversion of a single substrate to different terpene products [35]. Internal TPS can catalyze FPP molecular carbon chain cyclization to form a ring of different terpenoids or intermediates, through various enzymatic modification, such as isomerization, hydroxylation, oxidation, and reduction to form different structural and functional terpenoid derivatives.

DON biosynthesis requires a series of oxidation-reduction reactions. The expression level of FAD monooxygenase (FADMO) in Fg1 was significantly higher than that in Fg2. FADMO known as a hydroxylase, is an oxidoreductase based on blood-red knot sulfur salt, which is an active center for the catalyzation of oxygen atoms. It has been shown that FADMO produced by *F. verticillioides* can catalyze Baeyer-Villiger oxidation of steroidal compounds to generate lactone compounds. FADMO is also involved in the formation of various important substances, which is closely related to secondary metabolism, in fungi.

A total of 898 DEGs (|log2 (Fold Change)| > 1) were noted, of which 563 (62.7%) were up-regulated, and 335 (37.3%) were down-regulated. The result indicated significant differences in gene expression profile of Fg1 and Fg2, thereby revealing their phenotypes considering DON-production. The findings revealed eight genes related to DON biosynthesis, and their expression levels were verified by qPCR. The expression level of 7 out of 8 genes was higher in Fg1 than in Fg2. All these genes belonged to *TRI* cluster which is responsible for DON synthesis in *F. graminearum*. Most of the *TRI* were poorly expressed in Fg2 demonstrating the reason for no DON production in Fg2. In addition, STPK and STPK-Cek1 belonged to MAPKs that positively regulate DON biosynthesis in *F. graminearum* [24]. Both of them were significantly up regulated in Fg1. The transcription factor, Atf1, negative regulates DON synthesis, and Van-Nguyen et al. [28] reported that DON production in *FgAtf1*-deficient mutants was 5-fold more than that in wild-type strains in vitro. Our results indicated that DON biosynthesis was regulated not only by *TRI* genes, but also by protein kinases, in Fg1.

## 4. Conclusions

In conclusion, owing to poor expression level of the genes involved in localization at the host, Fg1 requires synthesis of DON to colonize the host. The supply of raw materials and substrates during DON biosynthesis was activated in Fg1 leading to biosynthesis of FPP, which is a precursor of DON. Most of the genes involved in DON biosynthesis in Fg1 were activated, which affected the rate of DON production. Finally, the MAPKs and transcription factors, which were involved in DON synthesis, were significantly differentially expressed in Fg1 and Fg2. In conclusion, all of the above factors determine the different capabilities of DON-production in Fg1 and Fg2.

## 5. Materials and Methods

### 5.1. Strains, Media and Culture Conditions

Strains were acquired by the following method. Briefly, the wheat with Fusarium head blight was collected and treated with 0.1% sodium hypochlorite solution for 1 min, and washed three times with sterile water. The collected wheat was inoculated on Bengal red medium (BRM: 31.6 g Bengal red (Sangon Biotech Co., Shanghai, China); and add deionized water up to 1 L). Seven days after incubation at 25 °C, the spores of the growing fungal strain were detected using a microscope. *Fusarium* spp., which show presence of the sickle conidia was inoculated to new BRM until a pure culture was obtained. *Fusarium* spp. was then cultured on potato dextrose agar (PDA: extract of 200 g boiled potatoes; 20 g glucose (Sangon Biotech Co., Shanghai, China); 20 g agar (Sangon Biotech Co., Shanghai, China) add deionized water up to 1 L) at 25 °C for 7 days, then the culture was maintained at 4 °C. *Fusarium* spp. was grown on PDA medium at 25 °C for 7 days before use.

### 5.2. Screening of F. graminearum with Specific PCR

The mycelium of *Fusarium* spp. growing on PDA was collected at 7 days after incubation at 25 °C and was ground to fine powder using nitrogen. Genomic DNA was extracted from all the strains using the Ezup Column Fungi Genomic DNA Purification Kit (Sangon Biotech, Co., Shanghai, China) according to the manufacturer’s instructions. *F. graminearum* specific primers—Fg16F (forward): 5′-CTCCGGATATGTTGCGTCAA-3′ and Fg16R (reverse): 5′-GGTAGGTATCCGACATGGCAA-3’ [36] were used to identify the seven strains. PCR was performed as follows: 2 μL DNA template (~50 µg), 2.5 μL 10X rTaq PCR buffer (Mg^2+^ plus), 2.5 μL dNTPs (2.5 μM each), 2.5 µL each primer (0.5 µM each), 0.2 μL rTaq DNA polymerase (TAKARA, Tokyo, Japan), and sterile water up to 25 μL. The conditions for the PCR amplification assay were: 94 °C for 5 min; 32 cycles at 94 °C for 1 min, 54 °C for 1 min, and 72 °C for 1 min; and 72 °C for 10 min. The ITS region of the two strains, Fg1 and Fg2, which were screened using the specific primer pair, Fg16F and Fg16R, were then identified by primers ITS1: 5′-TCCGTAGGTGAACCTGCG-3′ and ITS4: 5′-TCCTCCGCTTATTGATATGC-3′ [37]. PCR was performed as mentioned above. The PCR results were determined using agarose gel (1% *w*/*v*) electrophoresis. The PCR product was sequenced and the sequences were subjected to BLAST analysis using NCBI nr database [38]. These sequences homologous to Fg1 and Fg2 were obtained. These homologous sequences were clustered by clustalW and phylogenetic tree was built using MEGA5.0.

### 5.3. DON Extraction and HPLC-UV Analysis

DON was extracted using ethyl acetate. Briefly, the strains, which were screened by the specific primer pair, Fg16F and Fg16R, were activated on Czapex yeast agar (CYA) (100 mL: 0.5 g Yeast extract, 0.1 g K_2_HPO_4_, 0.05 g MgSO_4_·7H_2_O, 0.05 g KCl, 0.001 g FeSO_4_, 3 g sucrose, 2 g agar) by incubation for 7 days at 25 °C. Then, the mycelia were inoculated into 50 mL 3% Mung Bean Soup (MBS) medium (1 L: 300 mL extract of 30 g boiled Mung Bean, 700 mL ddH_2_O) at 25 °C, and were shaking conditions at 180 rpm. At 3 days post inoculation, the spores were collected by centrifugation and washed three times with sterile water. One milliliter spores at a density of 1 × 10^5^ cells/mL were inoculated into 100 mL Czapex yeast broth medium (CYB) (100 mL: 0.5 g Yeast extract, 0.1 g K_2_HPO_4_, 0.05 g MgSO_4_·7H_2_O, 0.05 g KCl, 0.001 g FeSO_4_, 3 g Sucrose) and cultured at 25 °C, under shaking conditions at 180 rpm. At 15 days after incubation, the supernatant of the cultures was collected by centrifuging at 10,000 rpm for 10 min at 4 °C (Eppendorf, Hamburg, Germany). Then, ethyl acetate at three times the volume of the supernatant was added and mixed for 10 min. After standing for 10 min, the organic phase, which contained ethyl acetate was collected and the residue was extracted again with ethyl acetate. All the organic phase was collected and evaporated using vacuum rotary evaporator RE 2000E (Yarong, Shanghai, China) at 40 °C, 0.1 Mpa, 120 rpm. The residue was dissolved in methanol. DON was purified by deoxynivalenol immunoaffinity column IAC-030-3 (Pribolab, Qingdao, China) following the manufacturer's instructions. A high-performance liquid chromatography system Agilent 1260 (Agilent, Santa Clara, CA, USA) was used to quantify DON. The analytical column used was Zorbax, SB-C18 250 × 4.6 mm 5 μm (Agilent, Santa Clara, CA, USA). The UV detection wavelength was set at 218 nm and the column temperature was held at 35 °C. All the samples were filtered through a 0.22 μm Wondadisc NY organic filter (SHIMADZU, Kyoto, Japan) before use. Twenty microliter of sample was injected to the HPLC system. Acetonitrile and water (16:84, *v*/*v*) was used as the mobile phase and the flow rate was 1 mL/min. Data was collected and analyzed using Gilson Unipiont software 5.0 (Gilson, Inc., Middleton, WI, USA). 

### 5.4. Proteome Analysis

#### 5.4.1. Protein Sample Preparation

Each of three biological replicates were used to performed proteome analysis. As shown in DON analysis, 1 mL *F. graminearum* spore at a density of 1 × 10^5^ cells/mL was inoculated into 100 mL CYB and incubated at 25 °C, under conditions at 180 rpm. After 15 days, the mycelia were filtered using 100 mesh sieve, washed thrice with cold distilled water, and centrifuged at 10,000 rpm for 15 min (4 °C) every time. The mycelia were collected and then ground into fine powder by mortar using nitrogen. Thereafter, the powder was transferred and 10 mL protein extraction buffer (10 mM Tris-HCL, 1 mM EDTA, pH 8.0), 1 mM PMSF, 50 μg RNase A and 200 μg DNase were added. The mixture was oscillated and mixed well and then incubated in ice for 30 min (the mixture was shaken every 10 min). The samples were centrifuged at 10,000 rpm for 15 min (4 °C), and then the supernatant was collected. Three volumes of 20% trichloroaceticacid (TCA)/acetone that were pre-cooled at −20 °C were added to the supernatant, mixed and incubated at −20 °C for 12–16 h. Further, the sample was centrifuged at 15,000 rpm for 20 min (4 °C) and the pellets was collected. The pellets was washed three times with acetone (pre-cooled at −20 °C) and centrifuged at 15,000 rpm for 10 min (4 °C) every time. The pellets was air-dried on ice, and solubilized in lysis buffer (2 M thiourea, 7 M urea, 4% (*w*/*v*) 3-[(3-Cholamidopropyl)dimethylammonio] propanesulfonate (CHAPS) (Bio-Rad, Hercules, CA, USA), 18 mM dithiothreitol (DTT) and 0.5% Ampholyte (Bio-Lyte) (*v*/*v*, pH 3–10)). The protein concentration was determined by Bradford’s method [39], and, the protein samples were stored at −80 °C until use.

#### 5.4.2. Two-Dimensional Gel Electrophoresis and Image Analysis

Two-dimensional gel electrophoresis was performed as described by Wang et al. [40] with some modifications. A 24-cm IPG strip (GE Healthcare, Piscataway, NJ, USA) was used to perform the first dimension electrophoresis. First, protein samples were diluted to 3 mg/mL using rehydration solution (2 M thiourea, 7 M urea, 4% (*w*/*v*) CHAPS, 18 mM DTT, 0.5% (*v*/*v*) Bio-Lyte (pH 3–10)). Four hundred and fifty microliters (1.35 mg) of the diluted protein sample was added to the IPG-box (GE Healthcare, Piscataway, NJ, USA) and the gel side of the strips was covered on the protein sample. After 14 h incubation at room temperature, the rehydrated strips were used to perform isoelectric focusing and were then equilibrated by two steps with two equilibration solution (50 mM Tris-HCl buffer, 6 M urea, 20% (*v*/*v*) glycerol and 2% (*w*/*v*) SDS supplemented with 2% (*w*/*v*) DTT, and 2.5% (*w*/*v*) iodoacetamide, respectively. During each step, the incubation was done under room temperature at 120 rpm for 15 min. The second dimension was electrophoresed on 12.5% polyacrylamide gel using an Ettan DALT System (GE Healthcare, USA). Marker protein was added to the ‘+’ side to evaluate the molecular mass of proteins. Electrophoresis conditions were as follows: 60 voltage for 1.5 h followed by 275 voltage for 4 h. After electrophoresis, the gels were visualized using Coomassie Blue stain (1 L: 450 mL methanol, 450 mL ddH_2_O, 100 mL glacial acetic acid and 2.5 g R250). The stained gels were scanned and analyzed using Image Master 7.0 analysis software (GE Healthcare, Piscataway, NJ, USA). Protein with an average fold change >2, *p*-value < 0.05, and exhibiting the same expression pattern among the three replicates were considered as significantly differentially expressed proteins.

#### 5.4.3. In-Gel Digestion

Differentially expressed proteins were collected from the gel, washed twice for 15 min using 350 μL distilled H_2_O, and destained with 100 μL 50 mM NH_4_CO_3_/50% acetonitrile. Ten millimolar DTT was added to the gel to reduce the proteins under 55 °C for 45 min. Then, 55 mM iodoacetamide was added to alkylate the protein at room temperature for 45 min. Acetonitrile (50%)/0.025 mM NH_4_HCO_3_ was added to wash the protein, which was further dried on ice. The dried proteins were incubated overnight at 37 °C in 10 μL 10 ng/mL trypsin (Sigma-Aldrich, St. Louis, MO, USA). The supernatant was collected for MS analysis.

#### 5.4.4. MS Analysis and Database Query

MS analysis was performed as described by Zhang et al. [41] and Zheng et al. [42] with some modifications. Each protein solution was mixed with an equal volume of matrix solution (70% acetonitrile, 0.1% trifluoroacetic acid, 10 mg/mL α-cyano-4 hydroxycinnamic acid). Mass spectra were analyzed using MALDI-TOF mass spectrometer (BrukerDaltonics, Bremen, Germany). Sequence query using peptide mass values and corresponding fragment peak lists were used to search for protein sequences against the NCBInr and Swissport databases using MASCOT version 2.3 software (Matrix Science, Franklin, UK) with the following search parameters: taxonomy, all series, allowed modifications, carbamidomethyl of cysteine (fixed), oxidation of methionine (variable), peptide tolerance, ±0.3 Da. The highest MOWSE score was only considered as the most probable identification, and was significant (*p* < 0.05) when protein scores were >88 (NCBInr) or 70 (Swissport).

### 5.5. Transcriptome Analysis

#### 5.5.1. RNA Extraction and Quality Test

*F. graminearum* was cultured in CYB as described above. At 15 days after inoculation, the mycelia were collected and fine ground to powder using a mortar and pestle with liquid nitrogen. Total RNA was extracted using the fungal total RNA isolation kit (Sangon Biotech Co., Shanghai, China) following the manufacturer's instructions. RNA degradation and contamination was monitored on 1% agarose gels and the RNA purity was detected by NanoPhotometer^®^ spectrophotometer (IMPLEN, München, Germany). RNA concentration was measured using Qubit^®^ RNA Assay Kit in Qubit^®^ 2.0 Flurometer (Life Technologies, Carlsbad, CA, USA). RNA integrity was assessed using the RNA Nano 6000 Assay Kit of the Bioanalyzer 2100 system (Agilent Technologies, Santa Clara, CA, USA). The samples that meet the requirements were used for the next steps.

#### 5.5.2. RNA-Seq Library Construction and Sequencing

Each one RNA sample of Fg1 and Fg2 was used to perform RNA-seq. Three microgram RNA of each sample was used for preparing libraries. Sequencing libraries were generated using NEBNext Ultra RNA Library Prep Kit for Illumina (NEB, Ipswich, MA, USA) following manufacturer’s instructions and four index codes were added to attribute sequences of each sample. Briefly, mRNA was purified from total RNA using poly-T oligo-attached magnetic beads (Life Technologies, Carlsbad, CA, USA), and then fragmented using divalent cations under elevated temperature in NEBNext First Strand Synthesis Reaction Buffer (5X). Random hexamer primer and M-MuLV Reverse Transcriptase (RNase H) was used to synthesize the first strand cDNA. DNA Polymerase I and RNase H was subsequently used to synthesize the second strand cDNA. Exonuclease/polymerase was used to convert remaining overhangs into blunt ends. NEBNext Adaptor were ligated to prepare for hybridization after adenylation of 3′ ends of DNA fragments. The library fragments were purified with AMPure XP system (Beckman Coulter, Beverly, MA, USA) for selecting cDNA fragments of preferentially 150~200 bp in length. cDNA (size-selected and adaptor-ligated) was incubated with 3 μL USER Enzyme (NEB, Ipswich, MA, USA) at 37 °C, 15 min, followed by at 95 °C for 5 min. Then PCR was performed with Phusion High-Fidelity DNA polymerase, Universal PCR primers and Index (X) Primer. PCR products were purified by AMPure XP system and library quality was assessed using Agilent Bioanalyzer 2100 system. The clustering of the index-coded samples was performed on a cBot Cluster Generation System using the TruSeq PE Cluster Kit v3-cBot-HS (Illumia) according to the manufacturer’s instructions. RNA-seq libraries were sequenced on an Illumina HiSeq 2000 platform to generate 125 bp/150 bp single-ended reads.

#### 5.5.3. Bioinformatics Analysis of RNA-Seq Data

Raw reads were pre-processed to obtain the clean reads by removing reads containing ploy-N, reads containing adapter and low quality reads from raw data. Q20, Q30 and GC content of the clean data were assessed, Table S1. The clean data with high quality was used to perform all the downstream analyses. TopHat v2.0.12 can generate a database of splice junctions based on the gene model annotation file, which was used to align the paired-end clean reads to the reference genome of *F. graminearum* PH-1 (taxid: 229533) [43]. HTSeq v0.6.1 was used to count the reads numbers mapped to each gene [44]. Then, the number of fragments per kilobase of transcript sequence per millions base pairs sequenced (FPKM) of each gene was calculated based on the length of the gene and reads count mapped to this gene. FPKM was used to estimate the gene expression levels [45]. edgeR program package was used to adjust the read counts with one scaling normalized factor for each sequenced library. DEGSeq R package (1.20.0) was used to analyze the differential expression in two samples. Benjamini and Hochberg method were used to adjust the *p* values [46]. |log2 (Fold change)| > 1 and corrected *p* < 0.005 were considered significantly differential expression. The gene expression level in Fg1 and Fg2 from transcriptome data was shown in Supporting Information, S1.

#### 5.5.4. GO and KEGG Pathway Enrichment Analysis

GOseq R package was used to perform GO enrichment analysis of DEGs [47]. GO terms with corrected *p* < 0.05 were considered significantly enriched. The statistical enrichment of differential expression genes in KEGG pathways was tested by the KOBAS software.

### 5.6. qPCR 

The RNA used for quantitative reverse transcription PCR (qRT-PCR) analysis was extracted as described above. RNA extraction was performed from independently generated Fg1 and Fg2 samples on CYB at 15 days after incubation at 25 °C, under shaking condition at 180 rpm. Primers for qRT-PCR were designed using the Primer 6 software and synthesized by Sangon Biotech. cDNAs were acquired by reverse transcription from 1 μg total RNA using PrimeScriptRT reagent Kit with gDNA Eraser (TAKARA, Tokyo, Japan) manufacturer’s protocol. qRT-PCR analysis was performed on ABI 7300 Real-Time PCR System (Applied Biosystems, Foster City, CA, USA) using the SYBR Premix Ex Taq II Tli RNaseH Plus (TAKARA, Tokyo, Japan) and following the manufacturer’s protocol. *EF1A* gene was used as an internal control to normalize the expression data [48]. qRT-PCR experiment was repeated three times, each sample having three technique replicates. The relative expression level of genes was calculated using the 2^−ΔΔCT^ method [49] and standard deviation was calculated between three biological replicates. The gene specific primers are listed in Supporting Information Table S3.

### 5.7. Statistical Analysis

The data were analyzed by the analysis of variance (ANOVA) using the statistical program SPSS/PC version II.x, (SPSS Inc. Chicago, IL, USA) and the Duncan’s multiple range test was used for separation of means. The statistical significance was applied at the level *p* < 0.05.

## Figures and Tables

**Table 1:** DON production capacity of *F. graminearum* strains.

Sample	DON (μg/g)
Fg1	1.02 ± 0.015
Fg2	0.00 ± 0
Fg3	0.86 ± 0.012
Fg4	0.24 ± 0.012
Fg5	0.43 ± 0.013
Fg6	0.59 ± 0.015
Fg7	0.76 ± 0.013

**Table 2:** Differentially expressed proteins in Fg1 and Fg2 by MALDI-TOF/TOF analysis.

Protein Spot	Protein Name	NCBI Accession	Mass	PI	Species	Score	Peptide Matches
1	lithostathine precursor	gi|45430003	19720	5.75	*Bos taurus*	446	6(4)
2	glyceraldehyde-3-phosph ate dehydrogenase	gi|475671174	36199	6.10	*Fusarium oxysporum*	297	4(2)
3	aldehyde reductase 1	gi|758198063	37477	5.83	*F. graminearum*	501	7(4)
4	glyceraldehyde-3-phosphate dehydrogenase	gi|758205105	32320	5.87	*F. graminearum*	147	3(0)
5	type i restriction endonuclease subunit m	gi|503927174	63891	5.79	*Pseudoxanthomonas spadix*	72	1(1)
6	regenerating islet-derived 3 alpha	gi|134024814	19560	5.25	*Bos taurus*	168	2(1)
7	hypothetical protein fpse_07721	gi|685864163	59116	6.21	*Fusarium pseudograminearum*	420	4(4)
8	glyceraldehyde-3-phosphate dehydrogenase	gi|758205105	32320	6.10	*F. graminearum*	163	3(1)
9	hypothetical protein fpse_10416	gi|685869551	18507	8.86	*F. pseudograminearum*	333	5(2)
10	superoxide dismutase	gi|758197546	24948	7.14	*F. graminearum*	556	4(3)
12	predicted protein	gi|302897541	23291	5.94	*Nectria haematococca* mpVI 77-13-4	202	2(2)
13	hypothetical protein fpse_03954	gi|758198681	33014	5.32	*F. graminearum* PH-1	114	1(1)
14	hypothetical protein fpse_00105	gi|758186649	33716	6.12	*F. graminearum* PH-1	372	4(3)
15	hypothetical protein fpse_05320	gi|685859361	32998	5.32	*F. pseudograminearum* CS3096	271	3(3)
16	78 kda glucose-regulated protein like protein	gi|477521896	70770	4.94	*F. oxysporum*	715	6(5)
17	elongation factor 2	gi|584132859	93373	6.34	*F. verticillioides* 7600	540	7(3)
18	pyruvate dehydrogenase e1 component subunit beta	gi|758201343	41784	6.07	*F. graminearum* PH-1	377	6(3)
19	hypothetical protein td95_000288	gi|802102353	29901	4.87	*Thielaviopsis punctulata*	137	1(1)
20	hypothetical protein fpse_01472	gi|685851669	53851	5.39	*F. pseudograminearum* CS3096	829	10(7)
21	hypothetical protein fg05_03462	gi|596545344	108299	5.69	*F. graminearum*	454	5(5)
23	hypothetical protein fpse_06578	gi|685861877	20437	7.02	*F. pseudograminearum* CS3096	611	6(6)
25	ornithine carbamoyltransferase	gi|749661859	34612	5.75	*Pseudomonas* sp. CB1	64	1(1)
26	islet-derived protein 3-beta-like isoform x1	gi|512910064	19965	6.70	*HeterocepHalus glaber*	88	2(0)
27	hypothetical protein fpsg_08677	gi|758195374	17923	5.46	*F. graminearum* PH-1	413	5(4)
29	hypothetical protein fpse_10576	gi|685869871	16463	7.78	*F. pseudograminearum* CS3096	255	3(3)
30	hypothetical protein fpse_07721	gi|685864163	59116	6.21	*F. pseudograminearum* CS3096	371	4(2)
31	peptidylprolyl isomerase (ec 5.2.1.8) a precursor	gi|2118328	24688	9.23	*Tolypocladium inflate*	235	3(2)
32	cyclophilin, cytosolic form	gi|642360	19495	9.23	*Tolypocladium inflatum*	227	2(2)
33	hypothetical protein fpsg_00105	gi|699040963	33716	8.64	*F. graminearum* PH-1	442	4(3)
34	prolyl-tRNA synthetase	gi|602541635	65726	5.11	*Mycobacterium mageritense* DSM 44476	64	1(1)
35	4-diphosphocytidyl-2-C-methyl-D-erythritolkinase	gi|329748844	34575	7.74	*SpHaerochaeta coccoides* DSM 17374	62	1(1)
37	terpenoid synthase 19	gi|15231879	69187	5.68	*Arabidopsis thaliana*	137	1(1)
39	putative ATP synthase beta mitochondrial precursor protein	gi|629659977	54945	5.46	*Eutypa lata* UCREL1	454	5(3)
44	hypothetical protein fg05_11228	gi|596542472	70636	5.11	*F. graminearum*	200	3(1)
46	hypothetical protein fgsg_05797	gi|758204059	63567	5.09	*F. graminearum* PH-1	570	7(3)
47	cathepsin D precursor	gi|148231809	43881	5.50	*Xenopus laevis*	84	1(1)
48	cyclophilin type peptidyl-prolyl cis-trans isomerase/cld	gi|770311810	18857	7.80	*Aspergillus parasiticus* SU-1	92	1(1)
49	hypothetical protein fgsg_02523	gi|758192157	18958	4.43	*F.graminearum* PH-1	241	3(2)
50	heat shock protein 70-1	gi|38325811	71195	5.02	*Nicotiana tabacum*	554	6(4)
52	FAD-binding monooxygenase	gi|703062904	43658	6.56	*Catenuloplanes japonicus*	75	1(1)
54	elongation factor 2	gi|758211940	93309	6.34	*F. graminearum* PH-1	645	6(4)
57	aldehyde dehydrogenase	gi|589108195	54084	5.93	*Trichoderma reesei* QM6a	246	3(3)
58	six-bladed beta-propeller	gi|573988893	86498	6.10	*Cordyceps militaris* CM01	64	1(1)
59	2,3-bisphosphoglycerate mutase	gi|189194371	57827	5.36	*PyrenopHora tritici-repentis* Pt-1C-BFP	87	1(1)
60	hypothetical protein fg05_02937	gi|596543275	64906	5.67	*F. graminearum*	661	8(4)
61	peroxidase/catalase 2	gi|758200916	88543	6.49	*F. graminearum* PH-1	661	8(4)
66	regenerating islet-derived protein 3 gamma	gi|351710837	19837	6.70	*HeterocepHalus glaber*	89	2(0)

Protein spot: the number of the protein spot on gel; Protein name: the protein name; NCBI accession: the gene ID in NCBI database; Mass: molecular mass of the protein; PI: the isoelectric point; Species: the species of the protein belonged to; Score: the Score of peptide matched to proteins; Peptide Matches: number of the peptide matched to protein.

## Supplementary Materials

The following are available online at www.mdpi.com/2072-6651/9/6/184/s1, Table S1: Summary of total RNA sequencing quality in Fg1 and Fg2, Table S2: The reads mapped to the reference genome of *F. graminearum* PH-1, Table S3: Primers used for verification of differentially expressed genes.
