# Supplementary material for: Screening of Deoxynivalenol Producing Strains and Elucidation of Possible Toxigenic Molecular Mechanism
Source: Toxins (Basel). 2017 Jun 1;9(6):184. doi: 10.3390/toxins9060184 (PMC5488034; doi:10.3390/toxins9060184)
Supplement: Supplementary file 1 [file toxins-09-00184-s001.pdf]

Supplementary Materials: Screening of Deoxynivalenol Producing Strains and Elucidation of Possible Toxigenic Molecular Mechanism

Xiangfeng Zheng, Xiaoli Zhang, Lina Zhao, Maurice T. Apaliya, Qiya Yang, Wei Sun, Xiaoyun Zhang and Hongyin Zhang

Table S1. Summary of total RNA sequencing quality in Fg1 and Fg2

Sample	Raw reads	Clean reads	Clean bases	Error rate(%)	Q20(%)	Q30(%)	GC (%)
Fg1	32447410	26363446	3.95G	0.02	96.09	90.26	52.40
Fg2	33489384	31301746	4.70G	0.03	95.90	88.59	52.74

Table S2. The reads mapped to the reference genome of *F. graminearum* PH-1

Sample_name	Fg1	Fg2
Total reads	26363446	31301746
Total mapped	21449066 (81.36%)	24434800 (78.06%)
Multiple mapped	199832 (0.76%)	37180 (0.12%)
Uniquely mapped	21249234 (80.6%)	24397620 (77.94%)
Reads map to '+'	10619448 (40.28%)	12204521 (38.99%)
Reads map to '-'	10629786 (40.32%)	12193099 (38.95%)
Non-splice reads	17467139 (66.26%)	19627198 (62.7%)
Splice reads	3782095 (14.35%)	4770422 (15.24%)

Table S3. Primers used for verification of differentially expressed genes

Gene ID	Gene description	Forward primer (5'to 3')	Revers primer (5'to 3')
FGSG_08811	EF1A	GGCTTTCACCGACTACCCTCCTC	ACTTCTCGACGGCCTTGATGACAC
FGSG_09266	HMG-CoA Synthase	ACTTCCCCAGCCAGTATGTT	CGGTAAGAGCGAAAGAGTAGAT
FGSG_09197	HMG-CoA reductase	CTCCCACCCAATCCACA	AGACACGGCTTCCTTCAA
FGSG_05912	Mevalonate Kinase	ATGAAACTGAACCGCAAGA	AAAGGGTGGTAACGTGTAGG
FGSG_09764	5′-Phosphomevalonate	CTGAGTGGGATGTTGAGGTT	TTGGAGTCTTCGGGGTTT
FGSG_06784	FPP synthease	TACCGTCAGGAGGGTGTTG	GGAAGGTGGTCTCGTGGAA
FGSG_03537	TRI5	TTTCCCACCGAGTATTTTCT	TAGGGTCTACCTTGAGCATCT
FGSG_03535	TRI4	TGTTGCTGGGATTTTCGC	CGTATTTATCGTGCATCTTGGA
FGSG_07896	TRI101	CTACACCCAAATCAGTCTCCT	TTTCCCTCGCTAATGCC
FGSG_03540	Tri11	TGCTTCCTCGGGGTCTT	CCACGAAATCTGGTCTGTCT
FGSG_03534	TRI3	CGGCAGCCAAGGATAAC	CCCGATAAACTTTCGACAACCGATT
FGSG_03533	TRI7	GAGAACGCCAAAGGAAAA	AAGCAATAACCCCAACGA
FGSG_03532	TRI8	GTCAAGGGCTCTTCGTTTC	CTGGGCGTGTTGGTCAT
FGSG_00469	STPK	CCATCTTCTCCTGACCGACTT	CACCAACTTCTGCTTGACAATG
FGSG_01312	Cek1	GGCTATCTACTACGGCTCTAACA	CCAGGTCTTCCATCCACTTGA
FGSG_10142	Atf1	GAACAACATCCGCCATCCAA	CGCATCGCTCATCGTAGAC
